# Duration of Immunity Induced after Vaccination of Cattle with a Live Attenuated or Inactivated Lumpy Skin Disease Virus Vaccine

**DOI:** 10.3390/microorganisms11010210

**Published:** 2023-01-13

**Authors:** Andy Haegeman, Ilse De Leeuw, Laurent Mostin, Willem Van Campe, Wannes Philips, Mehdi Elharrak, Nick De Regge, Kris De Clercq

**Affiliations:** 1 Sciensano, Scientific Directorate Infectious Diseases in Animals, Unit Exotic and Vector-Borne Diseases, Groeselenberg 99, B-1180 Brussels, Belgium; 2 Sciensano, Scientific Directorate Infectious Diseases in Animals, Experimental Center Machelen, Kerklaan 68, B-1830 Machelen, Belgium; 3 Sciensano, Scientific Directorate Infectious Diseases in Animals, EURL for Diseases Caused by Capripox Viruses, Groeselenberg 99, B-1180 Brussels, Belgium; 4 M.C.I. Santé Animale, Research and Development Department, Lot. 157, Zone Industrielle Sud-Ouest (ERAC) B.P: 278, Mohammedia 28810, Morocco

**Keywords:** lumpy skin disease, vaccines, duration of immunity

## Abstract

Vaccines have proven themselves as an efficient way to control and eradicate lumpy skin disease (LSD). In addition to the safety and efficacy aspects, it is important to know the duration for which the vaccines confer protective immunity, as this impacts the design of an efficient control and eradication program. We evaluated the duration of immunity induced by a live attenuated vaccine (LSDV LAV) and an inactivated vaccine (LSDV Inac), both based on LSDV. Cattle were vaccinated and challenged after 6, 12 and 18 months for LSDV LAV or after 6 and 12 months for the LSDV Inac. The LSDV LAV elicited a strong immune response and protection for up to 18 months, as no clinical signs or viremia could be observed after a viral LSDV challenge in any of the vaccinated animals. A good immune response and protection were similarly seen for the LSDV Inac after 6 months. However, two animals developed clinical signs and viremia when challenged after 12 months. In conclusion, our data support the annual booster vaccination when using the live attenuated vaccine, as recommended by the manufacturer, which could potentially even be prolonged. In contrast, a bi-annual vaccination seems necessary when using the inactivated vaccine.

## 1. Introduction

Lumpy skin disease virus (LSDV), a member of the genus Capripoxvirus of the family Poxviridae, is a double-stranded DNA virus of approximately 150 kbp and is the causative agent of lumpy skin disease (LSD) [1]. This disease is best characterized by the formation of nodules throughout the bodies of cattle and water buffaloes [2]. In addition to the typical nodules, other, more non-specific clinical symptoms, can be observed, including fever, enlarged lymph nodes, excessive salivation and eye and nasal discharge [3,4]. In addition to the effect on animal health, the disease also has a socio-economic impact, as it leads to a sharp drop in milk yield, infertility (temporary or permanent), trade restrictions, reduced market value due to damaged skins and abortion in pregnant cows [5,6,7,8]. During outbreaks, the morbidity rates can vary from lower than 10% up to 85% [4,9], and the mortality generally remains relatively low (<3%) but can be higher in rare cases [10,11].

Following the first detection of LSD in Rhodesia [12], the virus has followed a northward migration and arrived in Greece in 2015 [13]. Then, there were multiple outbreaks across the Balkan region the following year [14]. Simultaneously, the virus spread to the Russian Federation and was introduced to the Indian and Asian subcontinent in 2019/2020 [15]. Homologous live attenuated vaccines (LSDV LAV) have proven themselves as an efficient way to control and eradicate LSD in the field [16,17,18] and under standardized laboratory settings [19]. Furthermore, vaccination is not only beneficial from an animal health perspective but also for the financial returns upon vaccination [6]. However, notwithstanding these obvious benefits, these vaccines may induce some adverse reactions, such as swelling at the inoculation site, reduction in milk production and a Neethling response in rare cases, meaning that reluctance against vaccination persists among a number of farmers [19,20,21]. The recent developments of and promising results with inactivated vaccines against LSDV [22,23] could address a number of these points of concern, as they typically induce less side-effects. Another important aspect is knowing how long the protection lasts after administration of the vaccine. This is a vital component when designing a vaccination schedule (biyearly, yearly, etc.), which can guarantee the continued protection of the animals (or herd) against the disease. This is not only relevant for the health of the animals, but also has organizational [24,25] and financial [26,27] implications for the farmers. These factors are of importance for the national disease control agency and for the participating farmers, as they can influence the selection of vaccines. However, the amount of data currently available on the duration of immunity induced by capripox vaccines is very limited. This knowledge gap can be in part explained by the lack of specific serological tools able to differentiate between previous or subsequent exposure to vaccine or wild type stains [28]. This makes it difficult to determine the true duration of immunity under field conditions, especially in endemic regions, since reinfections of vaccinated animals could boost the immune response without inducing clinical symptoms. A single vaccination with a live attenuated sheeppox vaccine has been shown to protect vaccinated sheep against sheeppox for at least 1 [29], 2 [30,31] or even 4 years [32]. Data in relation to the protection against LSDV is scarce, however. A study by Ngichabe et al. (2002) [33] showed 2-year protection against LSD using an experimental bivalent vaccine against Rinderpest virus and LSDV. However, the animals were in paddocks in a field in Kenya, whereby exposure to vectors carrying LSDV cannot be excluded. The latter could provoke a natural booster effect during these 2 years. The low number of African Zebu (Bos indicus) included in this study (*n* = 4) was problematic, as (1) after experimental infection of Bos taurus, only 50% developed clinical disease [19,34,35]; (2) Bos indicus is less susceptible to LSDV than Bos taurus [6,36]. This demonstrates the need for information regarding the duration of immunity under controlled and standardized circumstances, notwithstanding the practical difficulties this entails. To address this knowledge gap, this study aimed to compare the durations of immunity offered by a live attenuated and an inactivated LSDV-based vaccine based on 6, 12 and 18 month of vaccination (LAV only) under controlled and standardized conditions.

## 2. Materials and Methods

### 2.1. Challenge Virus and Cell Line

Cells of the ovine testis cell line, OA3.Ts (ATCC-CRL-6546), were cultured in DMEM (Fisher Scientific, Belgium) supplemented with 10% fetal calf serum (FCS; Thermo Fisher Scientific;Merelbeke Belgium), fungizone (1 µg/mL; Thermo Fisher Scientific; Merelbeke, Belgium) and gentamycin (20 µg/mL; Fisher Scientific; Merelbeke, Belgium).

The LSDV strain LSD/OA3-Ts.MORAN, kindly provided by the Kimron Veterinary Institute, Israel and the Israeli Veterinary Services, was used as the challenge strain. It was propagated and titrated on OA3.Ts as described in Haegeman et al. (2021) [19].

### 2.2. Serological Analysis

Serum samples collected during the animal experiments were analyzed for the presence of anti-LSDV antibodies using the immunoperoxidase monolayer assay (IPMA) and virus neutralization tests (VNT), as described in Haegeman et al. (2020) [37]. Two different VNT methods were applied and are referred to as VNT 1 (titration of the test serum against 100TCID50 of a reference LSDV strain) and VNT2 (titration of a LSDV reference strain against the test serum) in the text. In addition to the in-house assays, a commercial ELISA, ID Screen^®^ Capripox Double Antigen Multi-species (Innovative Diagnostics, Grabels, France) was used as well.

Antibody titrations were carried out by using a two-fold serial dilution of the test sera. The antibody titer was expressed as the highest serum dilution that was positive.

### 2.3. Virus Isolation and Titration

Virus isolations and titrations were carried out as described by Haegeman et al. [19] with OA3.T cells. The virus titers were calculated using the method by Spearman–Kärber [38,39], whereby wells displaying one or more viral plaques were designated as positive.

### 2.4. Animal Trial

#### 2.4.1. Vaccines

Two vaccines were included in this study, namely, the LSDV-based live attenuated vaccine Lumpyvax (MSD Animal Health; Kempton Park, South-Africa) and a LSDV-based inactivated vaccine obtained from MCI (Santé Animale; Mohammedia, Morocco). Vaccination was carried out as instructed by the manufacturer. For the inactivated vaccine, a booster vaccination was performed 21 days after the first prime vaccination, whereas only a single application was carried out for the live attenuated vaccine.

#### 2.4.2. Experimental Animal Study Design

The animal experiments were carried out in the vector proof BSL3 facilities of Sciensano. All animals were 6-months old Holstein bulls upon introduction in the BSL3 stable and were free of BVD and IBR.

Due to practical limitations, it was decided to carry out two separate animal trials, whereby an unvaccinated group was included in each trial. As all animals, vaccinated and controls, were challenged at the same time within each trial, the animals were introduced to the stable at different time points depending on the type of vaccine (booster or single application) and the duration analyzed. An overview is presented in Table 1.

The challenge strain (LSD/OA3-Ts.MORAN; titer 6.5 TCID50/mL) was administrated as previously described by Haegeman et al. [19]. Briefly, 5 mL was injected intravenously (vena jugularis) in combination with 4 intradermal inoculations: 2 locations on both sides of the neck (250 µL per site). After the challenge, the animals were monitored and sampled for at least 21 days. Euthanasia was carried out between 21 and 34 days post-challenge (dpc).

All animal experiments were conducted according to the European Union and Belgian regulations on animal welfare in experimentation. The protocol was approved by the joined Ethical Committee of Sciensano, authorization number 20150605-01_EC_Dierproef aanvraag_LSDV_BMG_2015.

#### 2.4.3. Clinical Evaluation and Scoring

All animals were daily clinically evaluated during the complete duration of the animal trial (acclimatization, post-vaccination and post-challenge). These clinical observations were translated into points, as described in Haegeman et al. [19], and used to calculate an overall total clinical score. The animals were also checked for a local reaction at the vaccination site, conjunctivitis, nasal discharge and diarrhea.

#### 2.4.4. Sampling

The following sampling scheme for EDTA, clotted and heparinized blood was used: (1) once during the acclimatization period (between 3 and 5 days prior to vaccination); (2) on the day of vaccination but before the injection (0 days post-vaccination (dpv)); (3) 3 times a week during the post-vaccination period up to 21 dpv; (4) after 21 dpv, only clotted and heparinized blood was collected once a week or every other week until challenge; (5) on the day of challenge but before the injection (0 dpc); (6) on a daily basis from 5 to 15 dpc and every other day before and after this period. Biopsies were taken when nodules first appeared to confirm the presence of LSDV. At necropsy, 25 to 26 tissue and organ samples were collected per animal, as described in Haegeman et al. (2021) [19].

### 2.5. Pan Capripox RT-PCR

For the detection of the lumpy skin disease virus genome, a previously published pan Capripox real-time PCR panel [40] was used. This panel consists of three real-time PCRs and was used as follows: (1) An initial screening of all the samples was done with the D5R real-time PCR. If the Cp value of a sample was higher than 37, the sample was tested with both other real-time PCRs (namely, E3L and J6R). (2) If in a time-consecutive sample series, the status of the animal changed in the D5R PCR (negative to positive and vice versa), the E3L and J6R PCRs were applied as well.

### 2.6. PCR Assay for Differentiating Infected from Vaccinated Animals (DIVA)

Differentiation between LSDV vaccine and wild type LSDV genome was performed using the real-time DIVA PCRs described by Agianniotaki et al. (2017) [41].

### 2.7. IFNγ Release Assay

The secretion of interferon gamma, after the stimulation of heparinized blood with LSDV, was measured by the BOVIGAM^®^ 2G kit (Thermofisher; Belgium) and used as a measure of the induced cellular immune response. The applied protocol is based upon the manufacturer’s instructions and Parida et al. (2006) [42]. Briefly, heparinized blood samples were analyzed within 24 h after collection. The heparin tubes were gently inverted before blood was transferred (1.5 mL) to a 24 well plate in triplicate. This allowed each sample to be stimulated with: (a) 1× PBS (100 µL/well), as a negative control; (b) Pokeweed mitogen (100 µL/well at 160µg/mL dissolved in 1× PBS), as a positive control; and (c) LSDV strain LSD/OA3-Ts.MORAN (100 µL/well at 6.8 TCID50/mL). The stimulating agent was mixed with the blood by pipetting, after which the 24 well plate was incubated overnight at 37 °C. The plasma was collected after centrifugation at 500 g for 10 min at room temperature and stored at −20 °C until analyzed. The cut-off of the sandwich ELISA for positivity was set to 0.3. The latter was based upon testing more than 100 negative Belgian cattle. The OD values of the positive and negative controls were monitored over time to identify false positive and negative results. A response was classified as strong, medium or weak when the corrected OD (=OD virus—OD PBS) was >2, between 1 and 2 or <1, respectively.

## 3. Results

### 3.1. Unvaccinated Control Groups

#### 3.1.1. Clinical Observations and Scoring

Following the inoculation with a virulent LSDV, all animals of both control groups (*n* = 10) developed a fever, which spiked around 7 to 9 dpc. The highest body temperature measured was 41.1 °C. All animals at least reached a body temperature of 40 °C for a minimum of 1 day. One animal (R2 LSDV con2) was euthanized at 15 dpc for ethical reasons due to the severity of the LSDV-induced clinical symptoms. Following the initial spike, the fever remained present for a prolonged period (at least more than 9 days) in 6 out of 9 animals, and the temperature returned to normal after 2–3 days in the other 3 animals (Figure S1). The impacts on the other clinical parameters (such as feeding behavior, general health and prescapular lymph node enlargement) were closely linked to the observed body temperatures. The first nodules appeared between 6 and 8 dpc, coinciding with the fever spike, and this in 7 out of 10 animals (70%; LSDV con1 *n* = 3/5; LSDV con2 *n* = 4/5). The capripox status of these nodules was confirmed by real-time PCR (Cp values between 12.5 and 18.5) of biopsies taken from all 7 animals. In addition, virus isolation was performed with one of these biopsies. The presence of infectious virus in that sample was demonstrated, as the virus could be readily isolated in the first passage. Interestingly, all animals with a prolonged fever developed nodules, but others did not. The difference in clinical scoring between control animals with or without nodules became visible between 6 and 8 dpc (Figure S2).

#### 3.1.2. Virology and Serology

Viremia was detected by real-time PCR in all animals which had developed skin lesions (*n* = 7), and no viremia was detected in animals without any skin nodules (*n* = 3). The onset was between 5 and 6 dpc, and the animals remained positive until the end of the trial. The viremic pattern (onset, duration, height) was very similar for both control groups (Figure S3). The virus could readily be isolated on cell culture from blood samples collected at 8/9 dpc from the viremic animals (*n* = 7). The percentage of PCR-positive organs and tissues was clearly higher for animals that developed nodules/viremia (92%) compared to animals that had not (44%). The viral loads seemed to be higher in animals with nodules/viremia, since approximately 70% of the organs/tissues of the viremic animals had a Cp value of <37, whereas this was only 15% for the non-viremic animals (Table S1). Interestingly, LSDV could be easily isolated from three different muscles types (musculus masseter, quadriceps and trapezius) from a viremic control animal (R2 LSDV con2) and even the viral titers determined from the former two, namely, 10^3^ and 10^2.5^ TCID50/mL.

Seroconversion was first detected at 8 dpc in one animal, and all were seroconverted by 12 dpc using IPMA (Figure S4). In the commercial ELISA, first detection and complete conversion were after 14 and 21 dpc, respectively. No difference in seroconversion was seen between animals with and without nodules. Neutralizing antibodies were first detected at 14 and 16 dpc using VNT1 and VNT2, respectively. No complete seroconversion was seen with either method (78%) before the end of the trials (Table S2).

### 3.2. LSDV LAV-Vaccinated Groups

#### 3.2.1. Clinical Observations and Scoring

**Figure 1:**
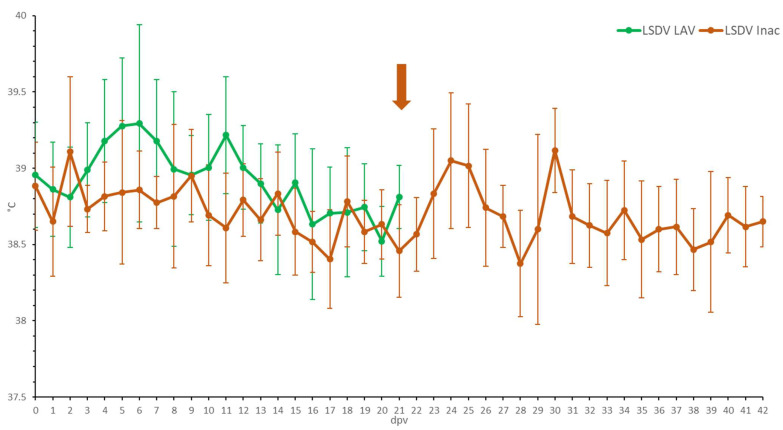
Averaged body temperatures of the LSDV LAV and the LSDV Inac animals after vaccination. Solid line: fever cut-off for consecutive days; arrow: moment of booster vaccination for LSDV Inac; standard deviation is shown as error bars.

Following vaccination, elevated body temperatures (39.5 to 39.7 °C) were observed in 44% of the animals. An additional 28% of the animals developed fever with a spike as high as 40.6 °C around 6 dpv. Body temperatures returned to normal in all groups between 7 and 13 dpv (Figure 1). No or limited impact of the vaccination was seen on the feed uptake, general behavior and general health status in all animals. Local reactions were seen at the site of vaccination in 50% of the animals. This reaction ranged from medium (size between 1.1 and 5 cm) to very strong (size >10.1 cm). During the vaccination period, the individual total clinical score of each of the animals did not surpass 2.5 (data not shown). During the vaccination period, two animals had to be euthanized due to complications of leg injuries unrelated to the vaccination. The averaged total clinical scores can be found in Figure S5.

**Figure 2:**
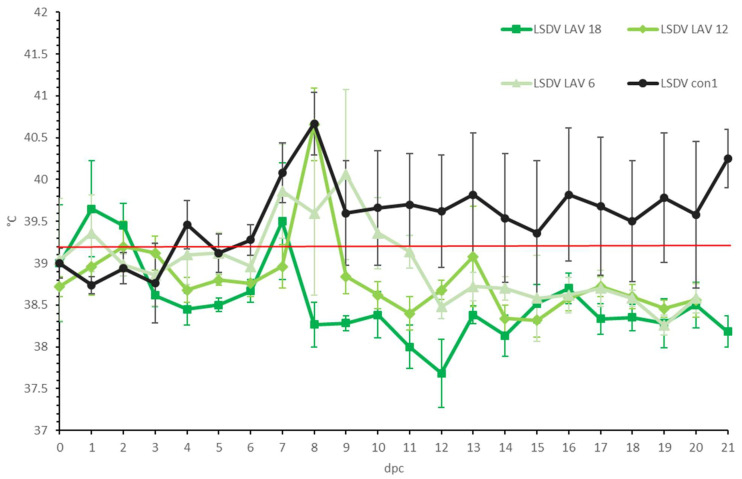
Averaged body temperatures of the LSDV LAV groups after challenge. For comparative purposes: the average of the control group LSDV con1 was added. Solid line: fever cut-off for consecutive days; standard deviation is shown as error bars.

**Figure 3:**
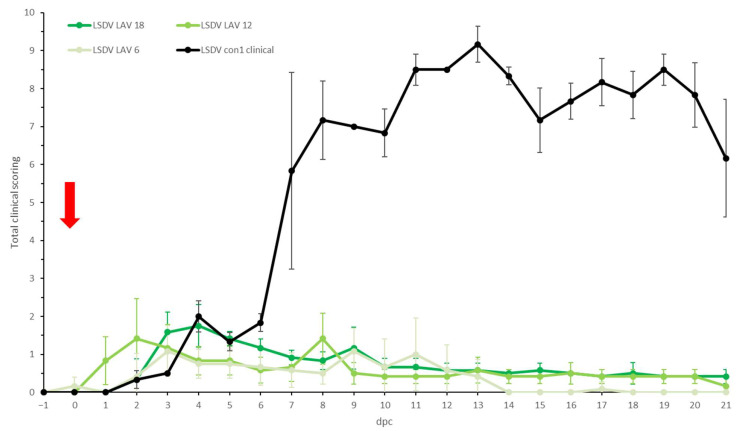
Averaged total clinical score of the LSDV LAV groups after challenge. For comparative purposes: the average of the clinical animals of control group LSDV con1 was added. The red arrow indicates the moment of challenge, and standard deviation is shown as error bars.

In all three LSDV LAV groups, elevated body temperatures were observed shortly after challenge in most animals. This was probably due to stress from the manipulation (Figure 2). A clear fever spike was seen between 7 and 9 dpc in 70% of the animals. However, the body temperature quickly returned to normal within 1 to 4 days. Reduced food uptake was observed between 2 and 9 dpc in 44% of animals (*n* = 7/16). No or limited impact of the LSDV challenge was seen on the general behavior and general health status. All animals were protected against other clinical signs, including the typical LSD nodule formation. The swellings across the four sites of inoculation were homogenous (small to moderate in size) and started to disappear from 7 to 9 dpc. The individual total clinical score of each of the vaccinated animals did not surpass 3.5. The averaged clinical score on the group level is represented in Figure 3.

#### 3.2.2. Virology

No vaccine viremia was observed in the LSDV LAV groups following vaccination, as only borderline-positive PCR results (Cp > 38) were obtained between 3 and 9 dpv (data not shown). These were probably due to the detection of the injected vaccine, given those tests’ close proximity in time to the vaccination itself. Similarly, no viremia was detected after challenge in all three groups, as again, only isolated, borderline-positive detections (Cp > 38) were obtained in some animals (*n* = 3).

In necropsy, only traces of the challenge virus genome were found in a very limited number of organs/tissues of the animals in all three groups, as determined by the pan capripox and the DIVA real-time PCR. The pattern of positivity was similar among groups challenged at 6-, 12- and 18-months post-vaccination. On average, between 7 and 9% of samples were weakly positive (Cp > 37) for LSDV (Table S3). Two exceptions were found in the 12-month group—namely, the skin samples taken at the site of challenge (Cp 36.2) and around the scrotum (Cp 33.3). The sample with the highest frequency of being positive in all three groups was the skin sample collected at the site of challenge (53%) and the normal skin sample (28%).

#### 3.2.3. Serology

**Figure 4:**
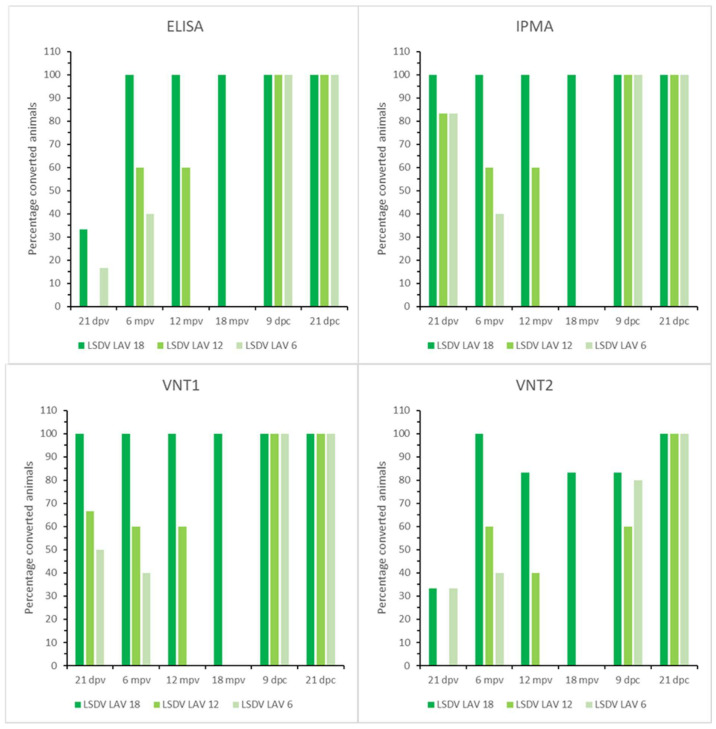
ELISA, IPMA, VNT1 and VNT2 results of the LSDV LAV groups; mpv: months post-vaccination.

The onset of seroconversion was seen at 7 dpv (IPMA), 11 dpv (ELISA and VNT1) and 16 dpv (VNT2). Complete seroconversion, as determined by IPMA, ELISA and VNT (1 and 2), was only seen in the 18-month group; it was between 83% and 40% in the other groups, depending on the serological test used (Figure 4). A decrease in antibody titers was seen for all vaccinated animals with the IPMA over time. However, animals remained antibody positive until the time point of challenge if the titer was 1/480 or higher at 21 dpv (Table S4). In those animals that became ELISA positive, the OD-values did not decrease over time and remained relatively stable until challenge. In addition, the decrease in neutralizing antibodies, as observed with VNT1 and 2, was less pronounced compared to the IPMA results. At the moment of challenge, 100%, 60% and 40% of animals in the 18-, 12- and 6-month groups, respectively, were positive according to IPMA, ELISA and VNT1; these numbers were 83%, 40% and 40% for VNT2. Following challenge, all animals were already found seropositive at 9 dpc by IPMA, ELISA and VNT1 (between 5 and 9 dpc); this took 21 dpc with VNT2 (Figure 4).

#### 3.2.4. Cellular Immunity (IFNγ-Assay)

**Figure 5:**
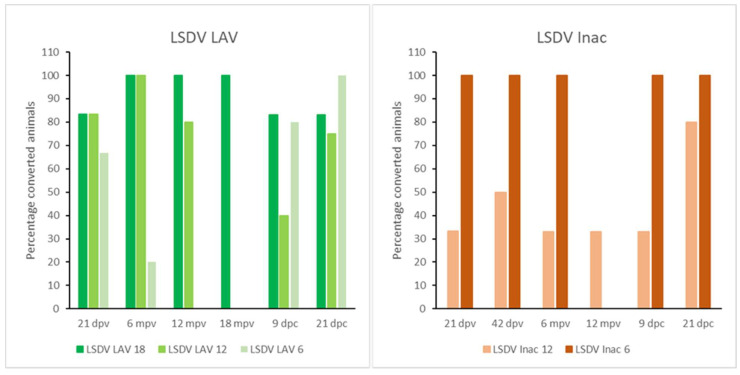
IFNγ results of the LSDV LAV and Inac groups; mpv: months post-vaccination.

The strength of the IFNγ response during the post-vaccination period varied over time, but all animals displayed a medium to strong response at some time (Figure S6). Almost all still showed a T-cell immune response at the moment of challenge in the 18- and 12-month groups (100% and 80% respectively). For the 6-month group, only 1 out of 5 animals had a IFNγ response at the moment of challenge (Figure 5), though 50% did a few days before.

### 3.3. LSDV Inac Vaccinated Groups

#### 3.3.1. Clinical Observations and Scoring

Compared to the LSDV LAV-vaccinated animals, elevated body temperatures (maximum 39.6 °C) were only seen in some animals (50%) for a short period following prime and booster vaccination (Figure 1). The animal R2 LSDV Inac 12 is somewhat of an outlier, as it had isolated fever spikes (up to 40 °C) 4 and 8 days after the booster. No negative impact of the vaccination was seen on the feed uptake, the swelling of the prescapular lymph nodes or general health status. No clear local reactions were seen at the site of vaccination after the first vaccination. After the booster vaccination, however, a small amount of swelling (0.5−2 cm) was seen in 25% of the animals. This swelling disappeared quickly after a few days. During the vaccination period, the total clinical score of all the animals did not surpass one. In general, the total clinical scoring of the LSDV Inac vaccinated animals was less than for the LSDV LAV-vaccinated animals (Figure S5).

**Figure 6:**
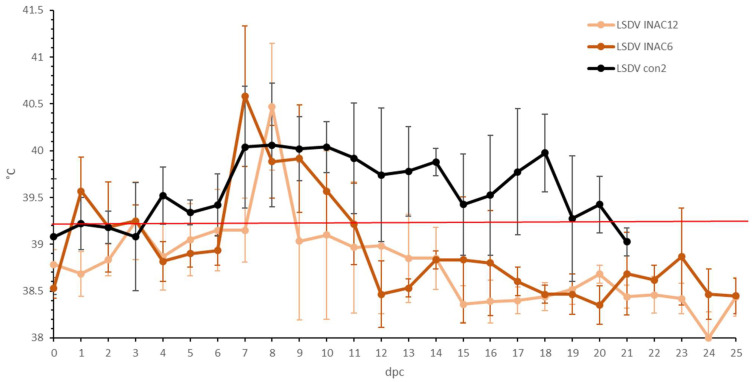
Averaged body temperatures of the LSDV Inac groups after challenge. For comparative purposes: the average of the control group LSDV con2 was added, Solid line: fever cut-off for consecutive days; standard deviation is shown as error bars.

**Figure 7:**
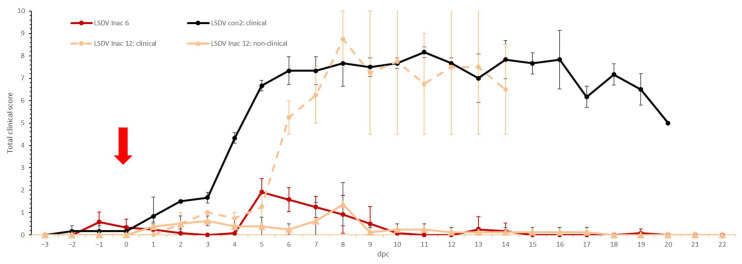
Averaged total clinical scores of the LSDV Inac groups after challenge. For comparative purposes: the average of the clinical animals of control group LSDV con2 was added. The red arrow indicates the moment of challenge, and standard deviation is shown as error bars.

A small rise in body temperature was seen around 1 to 3 dpc in the majority of the animals in both groups following the challenge, probably related to stress. A clear fever spike, with maximum temperatures between 40 and 41 °C, was seen between 7 and 9 dpc in almost all the vaccinated animals (92%) (Figure 6). The body temperature returned quickly to normal (9 to 12 dpc) in both groups, except for one animal in the LSDV Inac 12 group (R1), where the fever persisted at least until 14 dpc, at which the time the animal was euthanized due to ethical reasons, due to the LSDV. Reduced food uptake was observed in 75% of the vaccinated animals on the day of the fever spike. Food uptake returned quickly to normal except for one animal (R1 LSDV Inac 12). In the LSDV Inac 6 group, no other clinical symptoms were observed. In contrast, in the LSDV Inac 12 group, nodules appeared at 6 dpc on two animals (R1 and R2 LSD Inac 12). Virus could be easily isolated from biopsies taken from the noduli at 6 dpc from both animals. Both animals also had enlarged prescapular lymph nodes. The differences in the clinical picture and total scoring between the animals with and without nodules became visible at 6 dpc (Figure 7). During this period, the total clinical scores of all the animals which developed permanent nodules surpassed three and continued to rise afterward, whereas for animals that did not develop nodules or viremia, the total clinical score did not surpass three. This differentiating pattern between clinical and non-clinical animals was similar to that of the viremic control animals.

#### 3.3.2. Virology

Following vaccination, no vaccine viral genome was detected in any of the animals. After challenge, no LSDV viral genome was detected in any of the blood samples collected from the LSDV Inac 6 group. In the 12-month group, clear viremia was demonstrated by real-time PCR starting from 5 dpc in one animal (R1 LSDV Inac 12) which lasted until euthanasia at 14 dpc. Virus could readily be isolated from a positive blood sample taken at 7 dpc. In addition, a single positive blood sample was also obtained from another animal (R2LSDV Inac 12) at 9 dpc (Cp 35.8). By necropsy, only a limited number of organ/tissue samples (0 to 3) were positive per animal in the 6-month group, and most (8 out of 9) were borderline positive (Cp > 37; Table S5). In the LSDV Inac 12 group, a different picture was seen. This group can be divided in two. The positivity rates in the non-clinical animals reflect those from the 6-month group: 0 to 3 positive samples per animal, and the majority (75%) of the Cp values were above 37. One interesting exception was the skin sample at the location of challenge of one animal (R3 LSDV Inac 12), as the Cp value was 25. In the clinical animals, 92% of samples were positive for R1 LSDV Inac 12 and 50% for R2 LSDV Inac 12; 67% and 54%, respectively, of them had a Cp value lower than 37. Additionally, virus could be isolated from the inguinal lymph node and epididymis samples taken during necropsy from R1 LSDV Inac 12 animals.

#### 3.3.3. Serology

**Figure 8:**
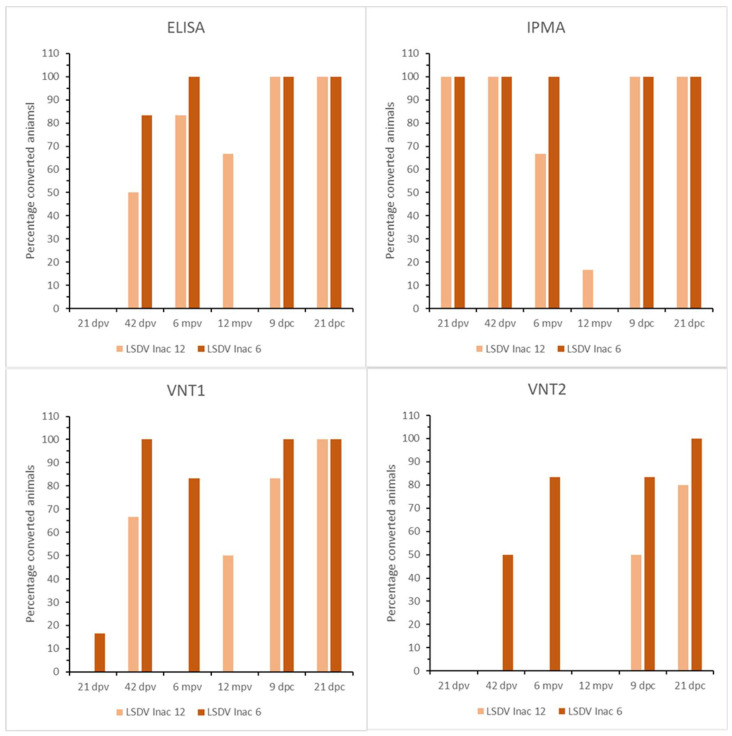
ELISA, IPMA, VNT1 and VNT2 results of the LSDV Inac groups.

The earliest onset of seroconversion was seen at 9 dpv by IPMA, at 28 dpv by ELISA and VNT2 and at 21 dpv by VNT1. While complete seroconversion was detected before booster vaccination at 21 dpv by IPMA, only 8% of the animals were positive by VNT1. A boosting effect on IPMA antibody titers was seen after the booster vaccination in the 6-month group, whereas this was absent in the 12-month group (Table S6). A similar difference was seen between the VNTs and ELISA. In the 6-month group, 100% and 83% of the animals were positive in VNT1 and VNT2, respectively, whereas only 67% and 0% were for the 12-month group. Although 100% seroconversion was seen with ELISA in both groups, the amount of induced antibodies was lower based on the S/N values in the 12-month group. Similarly to the LSDV LAV groups, a decrease in IPMA antibody levels over time was seen in both LSDV Inac groups. In general, the S/N values remained more stable in the ELISA, with a single exception (R3 LSDV Inac 12). In addition, 4 of 6 animals in the LSDV Inac 12 group continued to float around the cut-off until the end of the trial. At the moment of challenge, the positivity rate in the 12-month groups was 17%, 67%, 50% or 0% based on IPMA, ELISA, VNT1 and VNT2, respectively; it was found to be 100%, 100%, 83% or 83% for the 6-month group. Upon challenge, all negative animals quickly seroconverted; 100% seroconversion was seen at 6/7 dpc (IPMA, ELISA) or 12/14 dpc (VNT1, VNT2) (Figure 8).

#### 3.3.4. Cellular Immunity (IFNγ-Assay)

After the prime vaccination, 67% of all the vaccinated animals showed an IFNγ response, although this was transient in the majority of these animals (62.5%). Similarly to the antibody response, a stronger CMI response was observed in the 6-month group after the booster compared to 12-month group (Figure S7). This was translated into 100% of the animals being reactive to stimulation at the moment of challenge in the 6-month groups. Only two animals were reactive in the 12-month group. (Figure 5).

## 4. Discussion

Vaccines are an important tool for fighting pathogens, but their sustained use is tightly linked to several key characteristics, including duration of immunity. Therefore, it is not surprising that its determination is included in Regulation (EU) 2019/6, adopted on 22nd of January 2022 [43], and in the guidelines of the Committee for Veterinary Medicinal Products, EMEA/CVMP/682/99 [44]. However, not much is currently known about the duration of the protective immunity granted by the commercially available LSDV vaccines, and this lack was identified as an important knowledge gap by EFSA [45]. The current study was carried out in vector-proof BSL3 animal facilities in Belgium, which has an LSDV-free status under standardized conditions in a controlled vector-free environment. All the animals were 6-month-old Holstein bulls originating from an identical source. Although there is a gender bias, the impact on the presented results would have been minimal, as there is no statistical evidence showing a difference in LSDV susceptibility between male and female animals [46,47,48,49]). Due to the different post-vaccination periods (ranging from 6 to 12/18 months) the age of the animals at challenge varied between the groups. The exact impact of age on LSDV susceptibility is difficult to ascertain, as the data from the field can be confounded by other aspects, such as the difference in exposure to virus caused by time or management practices (for separate and different housing of calves). However, several studies show no age impact [47,50,51], and in another, the impact was limited to older adult animals (>4 years) [49]. All the animals in this study were between 1 and 2 years old when challenged, avoiding the extreme age groups (very young or adult animals). In view of the prolonged stay of the animal in the BSL3 facilities, adequate housing space was required from an ethical point of view, prompting the need to perform this study in two separate animal trials. In each trial, a control group was included, allowing us not only to compare both trials, but also to compare our trials with previous vaccination/challenge trials conducted using the same experimental design. The clinical picture (onset noduli, fever spike, etc.), including the virological (viremia, organ positivity, etc.) and serological (seroconversion) laboratory results, was very similar for the two trials in this study and also in line with the control group data presented by Haegeman et al. (2021) [19]. These data are also supported by the findings of Wolff et al. (2021) [52], Sanz-Bernardo et al. (2020) [53], Möller et al. (2019) [54] and Babiuk et al. (2008) [55]. This demonstrates that the challenge was successful and comparable, allowing the data in both trials to be compared to each other and to the data in those other studies. Of note is the finding of infectious virus in the muscles of one of the control animals. Kononov et al. (2018) [56] already reported the finding of infectious LSDV in muscles, but only in those adjacent and in direct physical contact to lesion sites. Although this could have been the case for the musculus masseter trapezius in this study, no lesions were seen in the area where the musculus quadriceps was sampled. This suggests that live virus can be present in muscles which are not close or in direct contact to lesions.

As seen with other LAV LSDV vaccines [19], a rise in body temperature at around 6 dpv was observed in the majority of animals vaccinated with Lumpyvax. A local reaction at the site of vaccination was noted during this study, as in other reports [57], though this was not the case in our previous one [19]. No additional side-effects were observed, not even a Neethling response, which is in agreement with its previously determined low prevalence of 0.1% [58] to 0.38% [16]. However, higher prevalences (up to 12.5%) have been reported [59], but this could have been due to a higher dose of vaccine being given. Only traces of the injected vaccine were found in the blood shortly after vaccination, which has also been demonstrated in the past for other viruses as well [60]. Seroconversion by IPMA after 21 days was 89%, which is comparable with previous data for this vaccine [19]. Interestingly, the five LSDV LAV-vaccinated animals, which had no antibodies at the time of challenge, were equally protected as the animals which were still positive. This would suggest an additional component in the host defense. A clue can be found in the IFNγ release data of these five animals. Four of them showed high IFNγ responsiveness after stimulation during the post-vaccination period. This substantiates the notion that humoral and cellular immunity play roles in the protection against LSDV, as has been previously postulated and reviewed [17,61]. Although the IFNγ T-cell-mediated response displayed an undulating pattern, blood samples collected after 18 months of vaccination remained reactive. These IFNγ data indicate strong stimulation of the cellular immunity by the live attenuated vaccine. This longevity pattern in the IFNγ T-cell-mediated immune response after vaccination with a LAV is not unique for LSDV and has been reported for other LAVs as well [62,63,64]. In the 18-month group, all animals remained antibody-positive until the day of challenge. The detection of LSDV antibodies for a prolonged period of time is in agreement with previous findings where LSDV antibodies were still detected after 40 [65] to 47 weeks [66]. The protection against a virulent challenge afforded by Lumpyvax was 100% in all three groups, as witnessed by the absence of clinical signs—except from a limited fever—such as nodule formation and viremia. This indicates that the duration of immunity of the Lumpyvax vaccine is at least 1.5 years.

The use of inactivated vaccines against capripox has been employed already with success for many years. They have been used in sheep to protect them against SPPV [67,68,69]. In contrast, it is only recently that their potential against LSDV has surfaced [22,23,70]. The inactivated vaccine used in this study was well-tolerated by the animals, as almost no side-effects were seen and were lesser than those of the live attenuated vaccine. The lack of PCR detections after vaccination further reinforces the safety of this vaccine and shows it is beneficial from a diagnostic point of view. The inactivated vaccine used in this study provided complete protection against challenge at 6 months post-vaccination, as demonstrated by the absence of viremia and clinical symptoms. At this time, almost all (92%; 11/12) of the vaccinated animals were still positive according to IPMA and ELISA. The averaged IPMA titer was similar to the one induced by the live attenuated vaccine. This level of seroconversion is higher than that reported by Hamdi et al. [22], which was 120 dpv, but the number of animals in that study was higher, and it was done in field-like conditions. In addition, the IFNγ T-cell mediated response was still very strong in the 6-month group at challenge. At 12 months after the vaccination with the inactivated vaccine, however, the protection was no longer complete, as witnessed by two vaccinated animals that became clinically ill upon challenge. The virus could be isolated from the blood, and nodules pose a potential threat for further transmission of the virus by vectors. Of note was the fact that one animal was only viremic at one sampling but became clinically ill. This shows a potential diagnostic risk, as it could have been missed easily on sampling, resulting in not detecting infected animals before the clinical manifestation of the noduli.

Of note was the difference seen in the immune responses elicited by the 12- and 6-month groups after the prime and booster vaccinations with the inactivated vaccine, and the same for LAV-vaccinated groups. The exact reason for this inconsistency is currently unknown. As mentioned above, the experimental setup (including personnel involved, stable used, etc.) was identical, as were the animals used (age, breed, farm of origin). The same batch of inactivated vaccine was used for both groups of LSDV Inac, wherefore this can be excluded as potential source of the variation. For the LAV groups, however, two batches of Lumpyvax were used. The difference was most pronounced between LSDV LAV 18 and the other two groups, but the same batch was used for the groups LSDV LAV 18 and LSDV 12, making it unlikely that different batches was the main driver. This is further supported by comparing this data with the data published for this vaccine by Haegeman et al. (2021) [19]. In this study, even another batch of Lumpyvax was used, and the onset of the immune response was comparable to the onset observed for LSDV LAV12 and 6. However, the seronegative animals in the LAV vaccine groups were all protected, though this was not the case for the inactivated 12-month group. At first glance, the difference in antibody positivity rate between the IPMA and ELISA (and also VNT1) is curious, as the former has been reported to be more sensitive [37]. This is probably due to the fact that the samples around this time point float around the detection limit of the different tests. This can be witnessed by the fact that: (1) 50% of the animals were still positive for IPMA at 10 months; (2) one animal became again borderline positive by IPMA at the moment of challenge when it had been negative on the four proceeding samplings; (3) at the moment of challenge, four animals had Elisa S/N values just above (*n* = 3) or just below (*n* = 1) the cut-off of the kit; animals had antibody titers by VNT1 equal to or above (*n* = 3) or below (*n* = 2) the 1/50 cut-off value; four animals had a neutralization index of 1.25 (just below the 1.5 cut-off for VNT close, or 2 at the moment of challenge.

## 5. Conclusions

In conclusion, it can be stated that the Lumpyvax vaccine gives complete protection for at least up to 1.5 year with limited side-effects. These findings indicate that the protection against LSDV after a primo vaccination is longer than the 12-month period mentioned by most LSD vaccine producers. It would be worthwhile to check whether this also holds true for other LAV vaccines and whether changes to the vaccination schedules could be proposed. The inactivated vaccine in this study gave complete protection for up to six months but did not protect all animals after one year. Therefore, a bi-annual vaccination strategy remains advisable for vaccines of this kind, as is also proposed for other diseases, such as foot and mouth disease (FMD) [71,72,73] and bovine ephemeral fever [74] and is advised in the WOAH FAQ regarding the use of LSDV inactivated vaccines (WOAH FAQ) [75].

## Figures and Tables

**Table 1:** Overview experimental setup. Duration of immunity is considered as the time between the last vaccination and the moment of challenge.

Type of Vaccine	Duration of Immunity Examined	Trial	Group Acronym	Animal Id	Nr. of Animals
Live attenuated vaccine	18 months	1	LSDV LAV 18	R1 to R6	6
	12 months	1	LSDV LAV 12	R7 to R12	6
	6 months	2	LSDV LAV 6	R13 to R18	6
Inactivated vaccine	12 months	2	LSDV Inac 12	R1 to R6	6
	6 months	2	LSDV Inac 6	R7 to R12	6
Controls					
Unvaccinated group		1	LSDV con1	R1 to R5	5
		2	LSDV con2	R1 to R5	5

## Data Availability

The main data presented in this study are available within the study itself and other data may be made available through contact with the corresponding author.

## Supplementary Materials

The following supporting information can be downloaded at: https://www.mdpi.com/article/10.3390/microorganisms11010210/s1. Figure S1: Body temperatures control animals averaged per trial. Figure S2: Total clinical scores of control animals. Figure S3: Viremia control groups, LSDV con1 and con2. Figure S4: IPMA and ELISA results of the control animals. Figure S5: Averaged total clinical score of the LSDV LAV and Inac groups after vaccination. Figure S6: IFNg results of the LSDV LAV 18 (a), 12 (b) and 6 (c). Figure S7: IFNg results of the LSDV Inac 12 (a) and 6 (b). Table S1: PCR results of organ and tissue samples of the unvaccinated control groups. Table S2: VNT1 and VNT2 results of both control groups. Table S3: real-time PCR results (pan capripox) of organ and tissue samples of the three LSD LAV groups. Table S4: Antibody titers of the LSDV LAV groups as determined by IPMA. Table_S5: real-time PCR results (pan capripox) of organ and tissue samples of the two LSD Inac groups. Table S6: Antibody titers of the LSDV Inac groups as determined by IPMA.
