# Supplementary figures and images for: Duration of Immunity Induced after Vaccination of Cattle with a Live Attenuated or Inactivated Lumpy Skin Disease Virus Vaccine
Source: Microorganisms. 2023 Jan 13;11(1):210. doi: 10.3390/microorganisms11010210 (PMC9864976; doi:10.3390/microorganisms11010210)

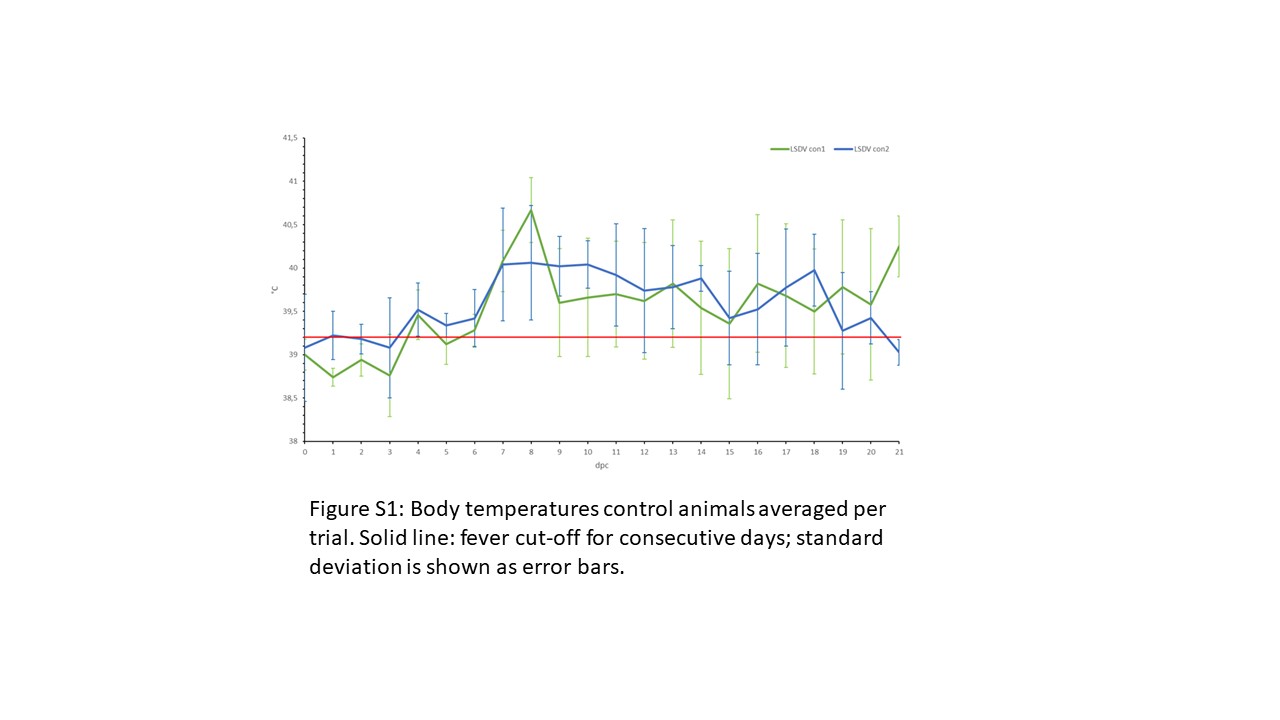

Supplement: Supplementary file 1 [file microorganisms-11-00210-s001.zip › Fig S1_300dpi.jpg]

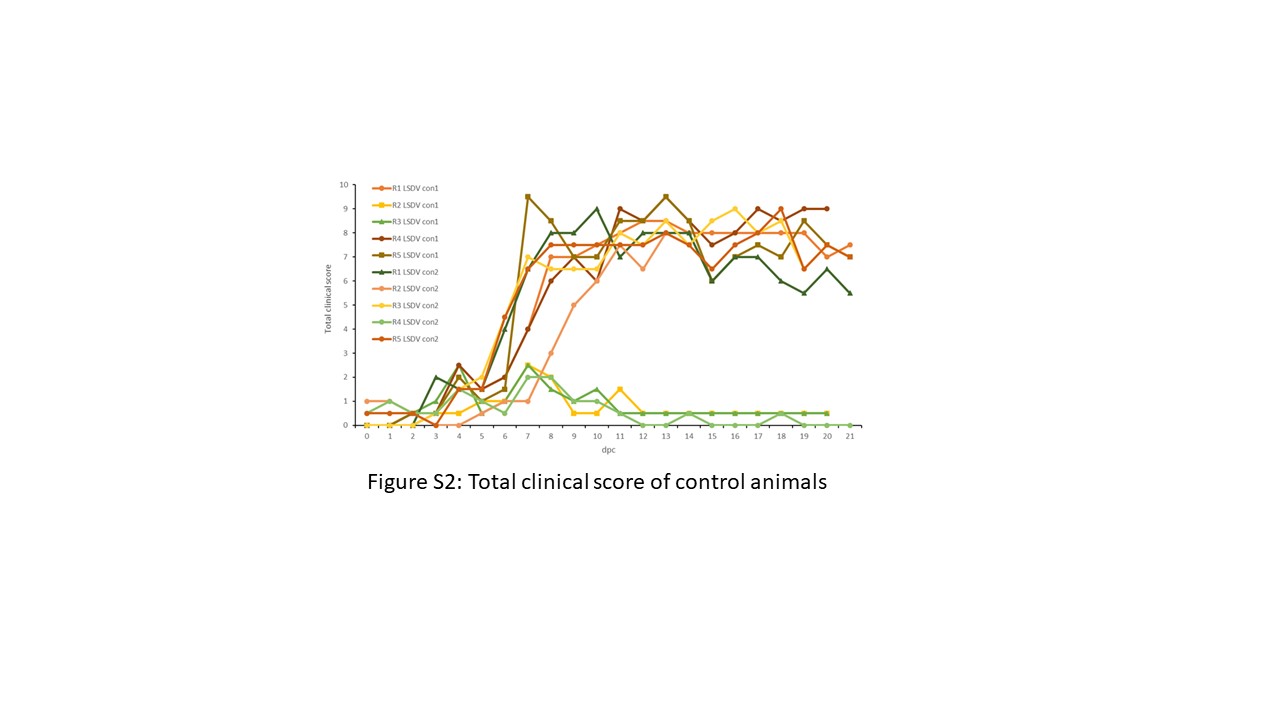

Supplement: Supplementary file 1 [file microorganisms-11-00210-s001.zip › Fig S2_300dpi.jpg]

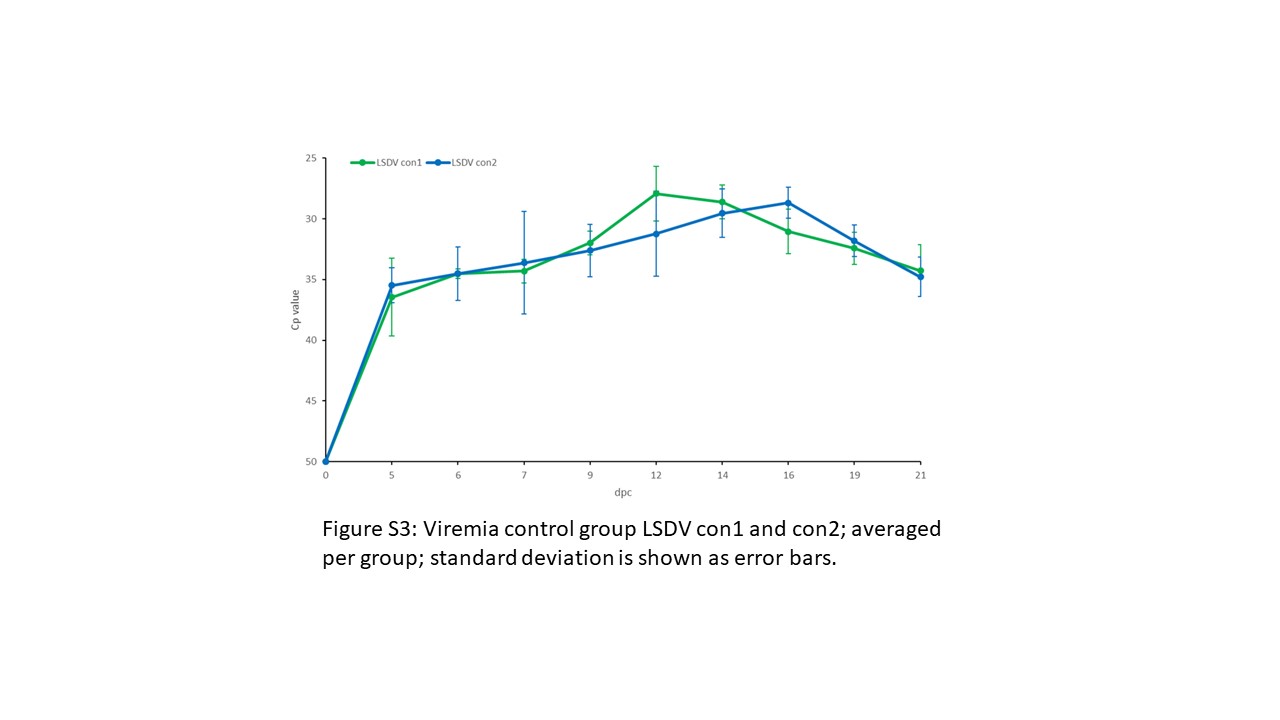

Supplement: Supplementary file 1 [file microorganisms-11-00210-s001.zip › Fig S3_300dpi.jpg]

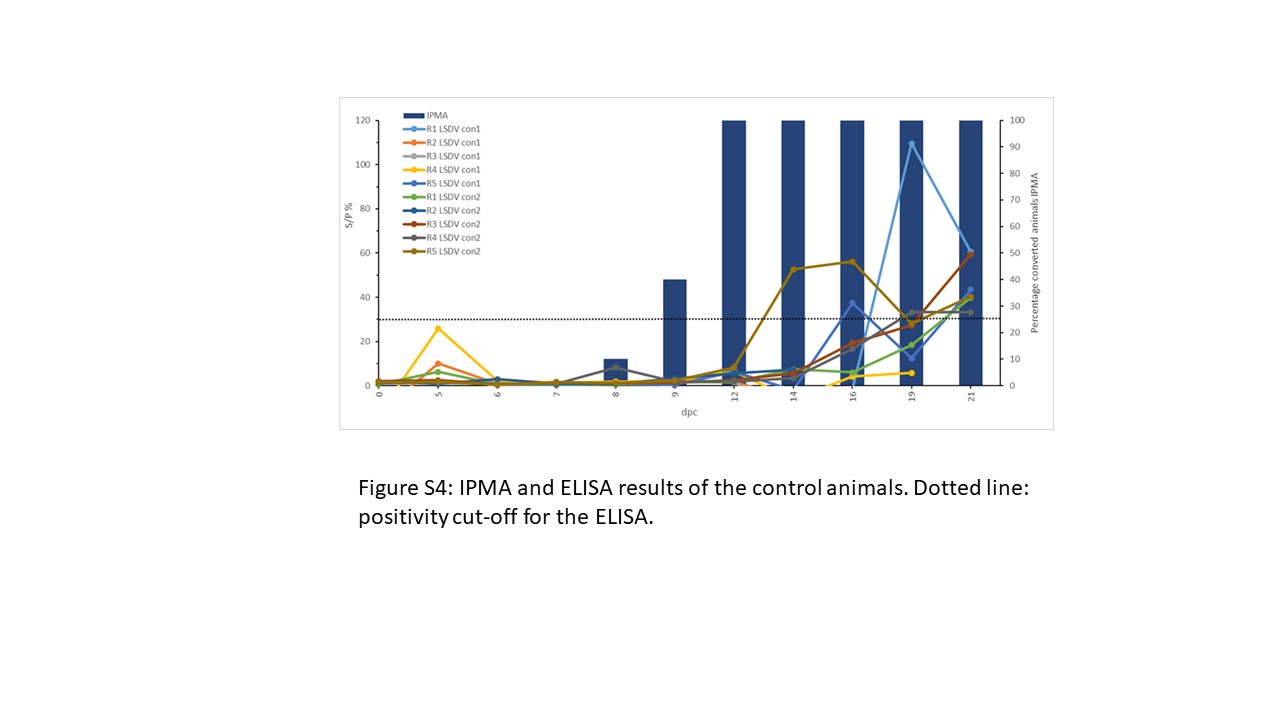

Supplement: Supplementary file 1 [file microorganisms-11-00210-s001.zip › Fig S4_300dpi.jpg]

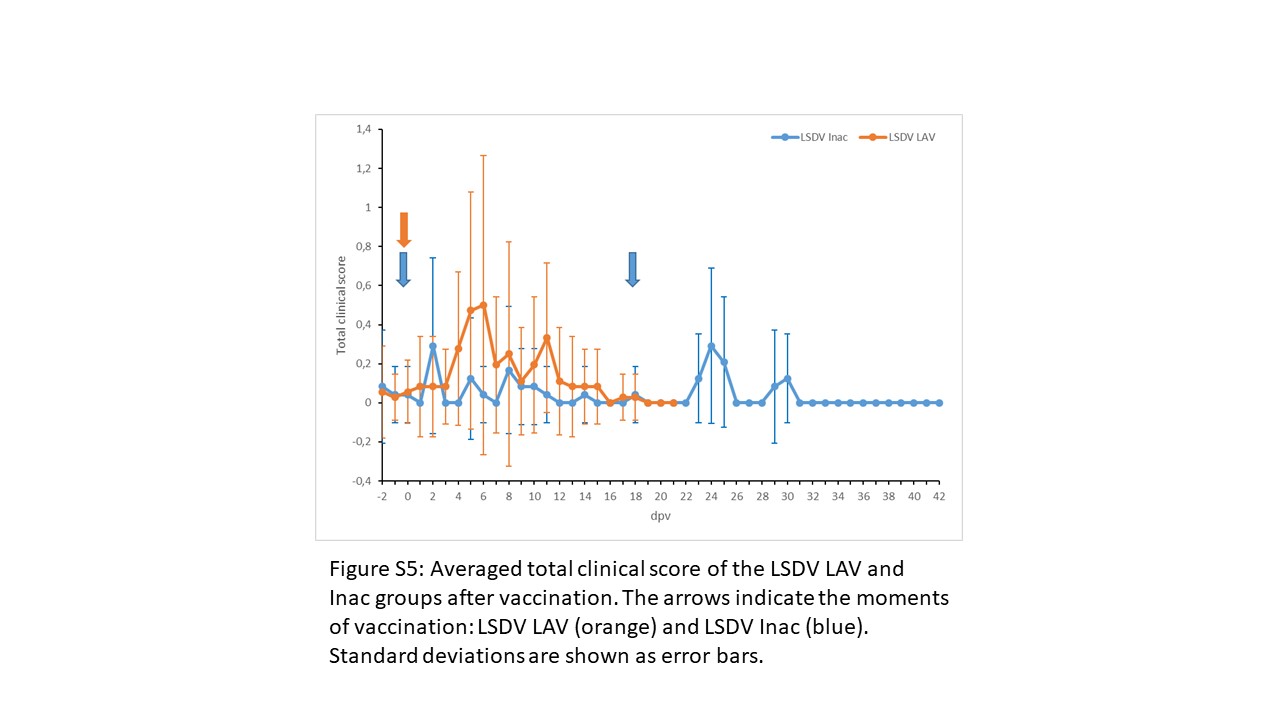

Supplement: Supplementary file 1 [file microorganisms-11-00210-s001.zip › Fig S5_300dpi.jpg]

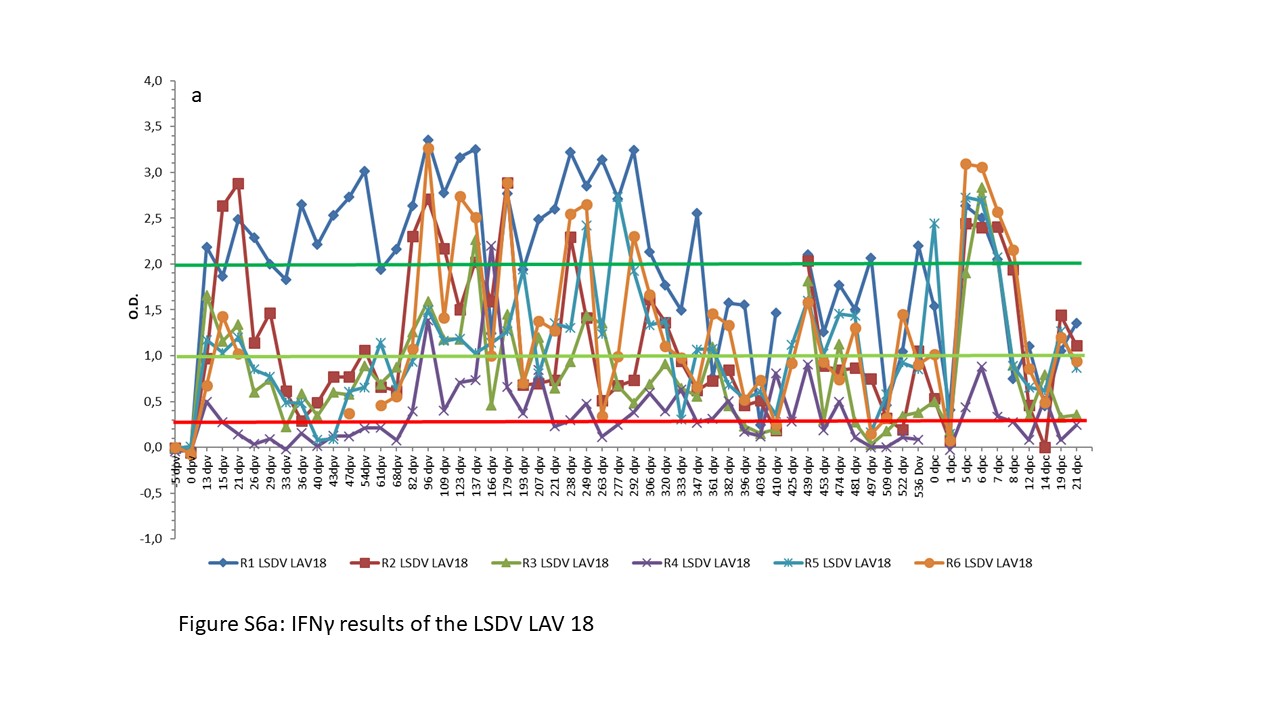

Supplement: Supplementary file 1 [file microorganisms-11-00210-s001.zip › Figure S6a_300dpi.jpg]

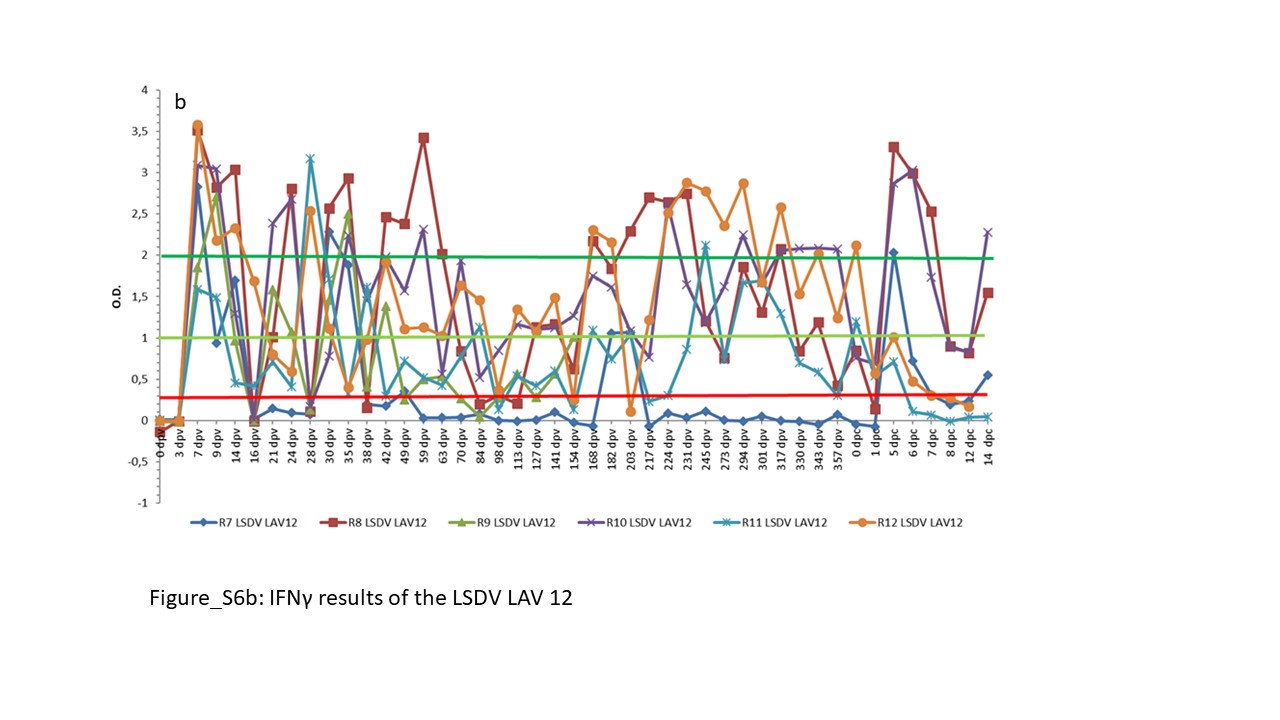

Supplement: Supplementary file 1 [file microorganisms-11-00210-s001.zip › Figure S6b_300dpi.jpg]

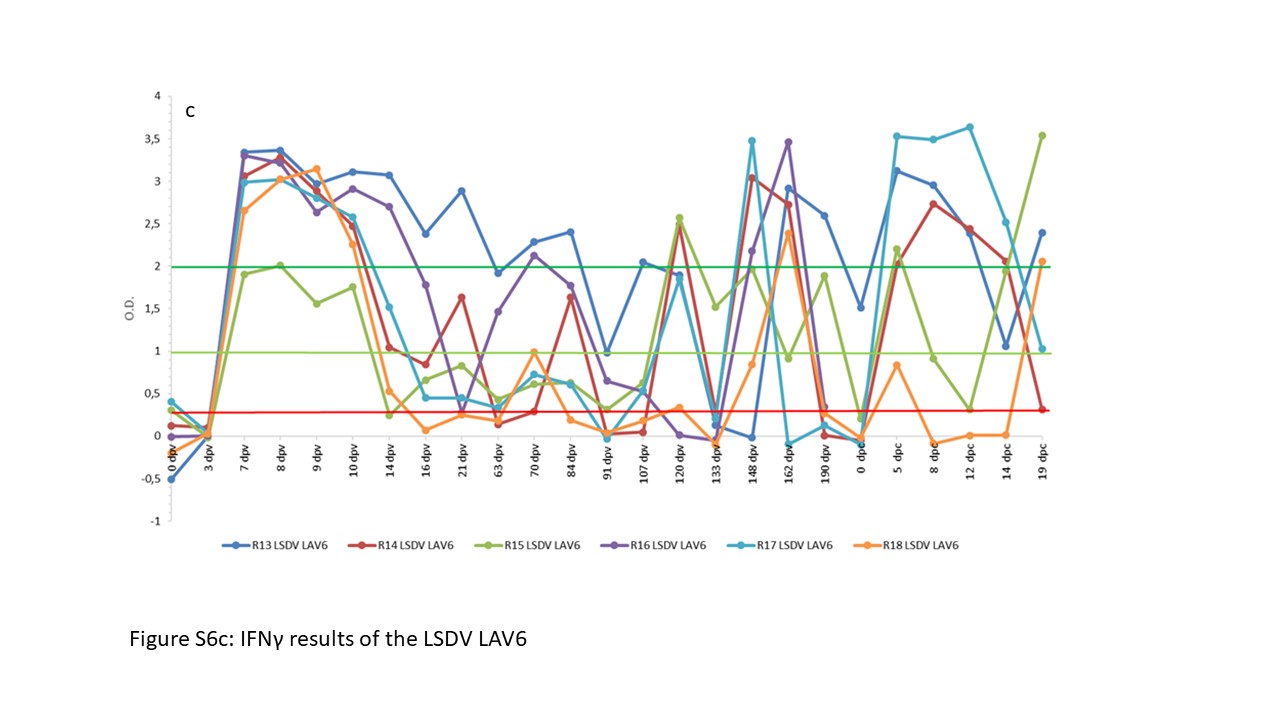

Supplement: Supplementary file 1 [file microorganisms-11-00210-s001.zip › Figure S6c_300dpi.jpg]

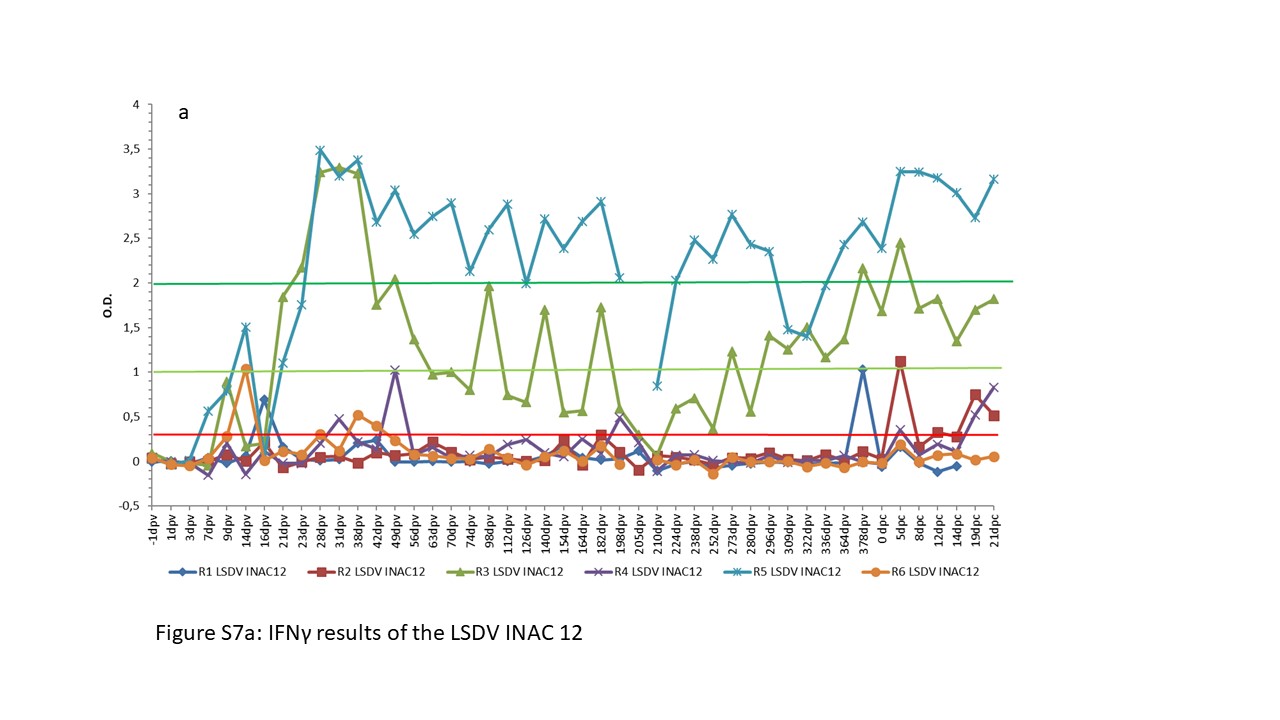

Supplement: Supplementary file 1 [file microorganisms-11-00210-s001.zip › Figure S7a_300dpi.jpg]

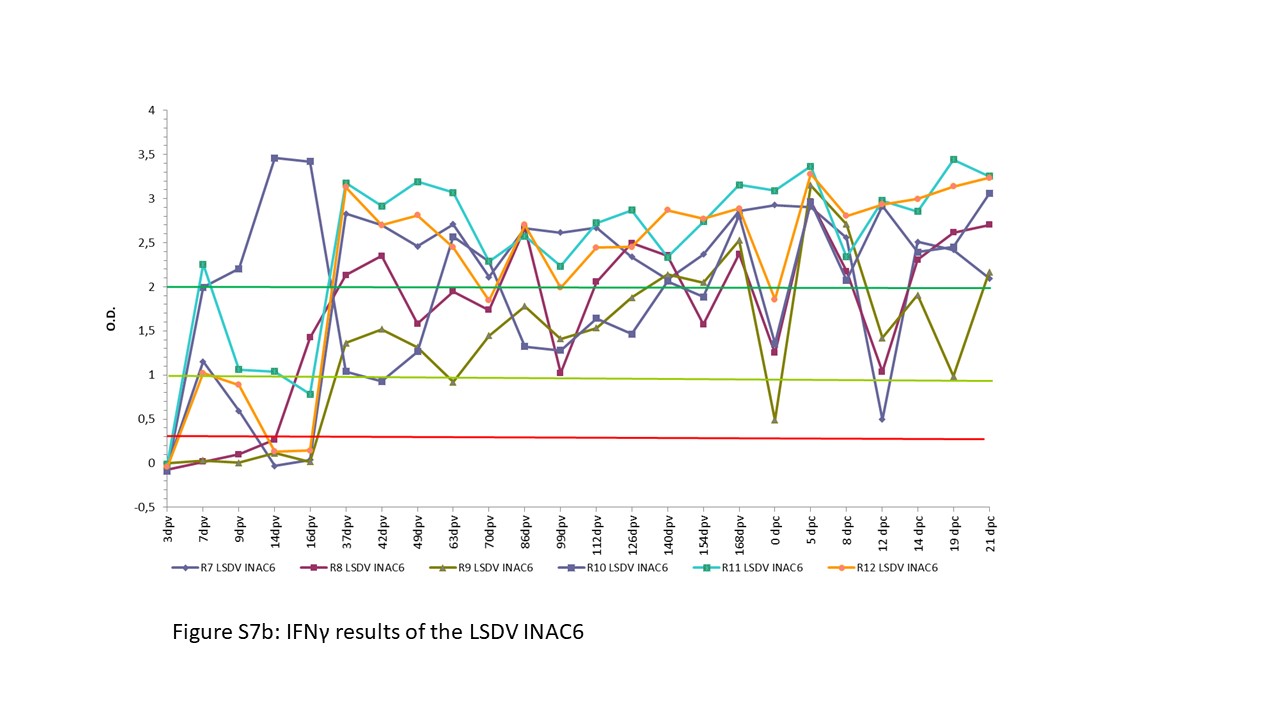

Supplement: Supplementary file 1 [file microorganisms-11-00210-s001.zip › Figure S7b_300dpi.jpg]

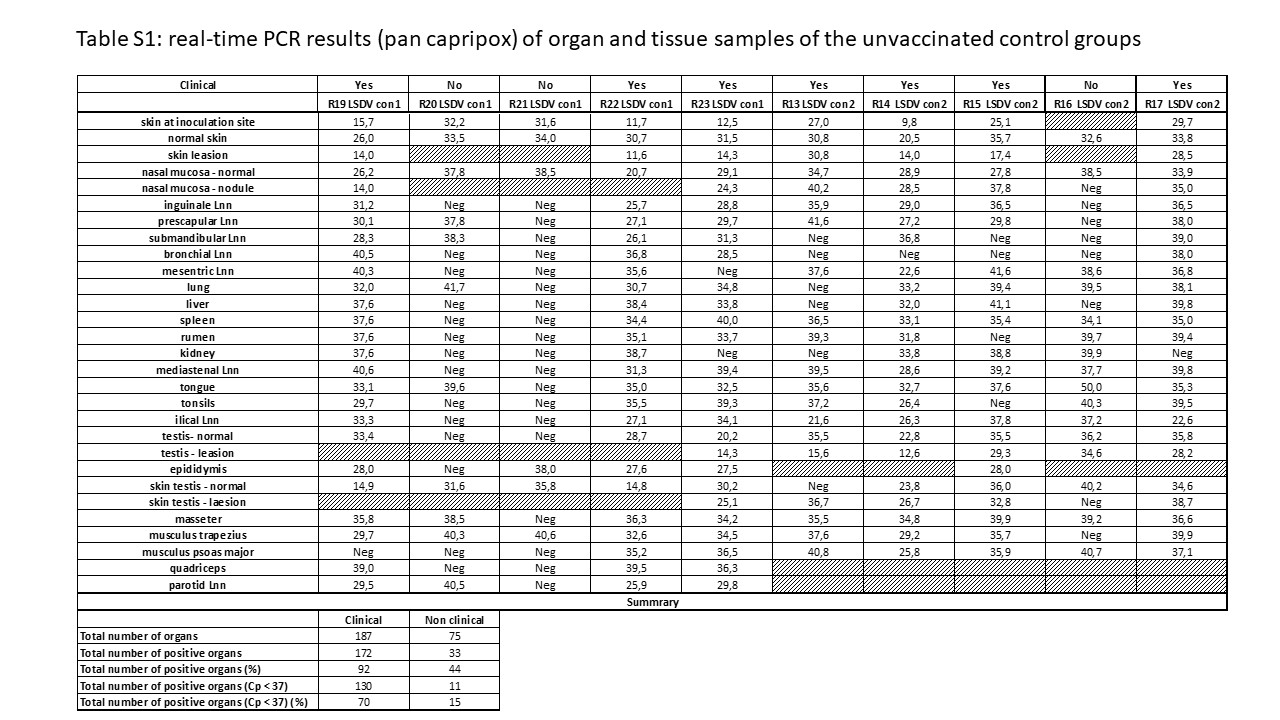

Supplement: Supplementary file 1 [file microorganisms-11-00210-s001.zip › Table S1_300dpi.jpg]

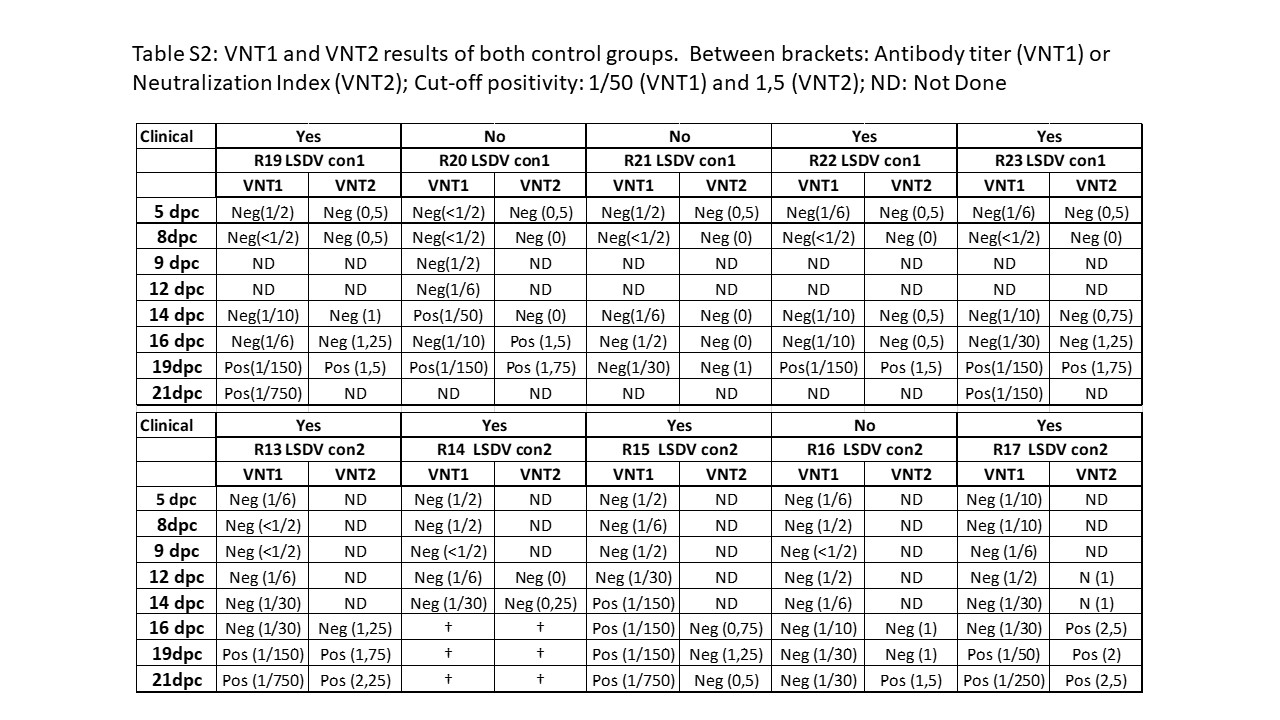

Supplement: Supplementary file 1 [file microorganisms-11-00210-s001.zip › Table S2_300dpi.jpg]

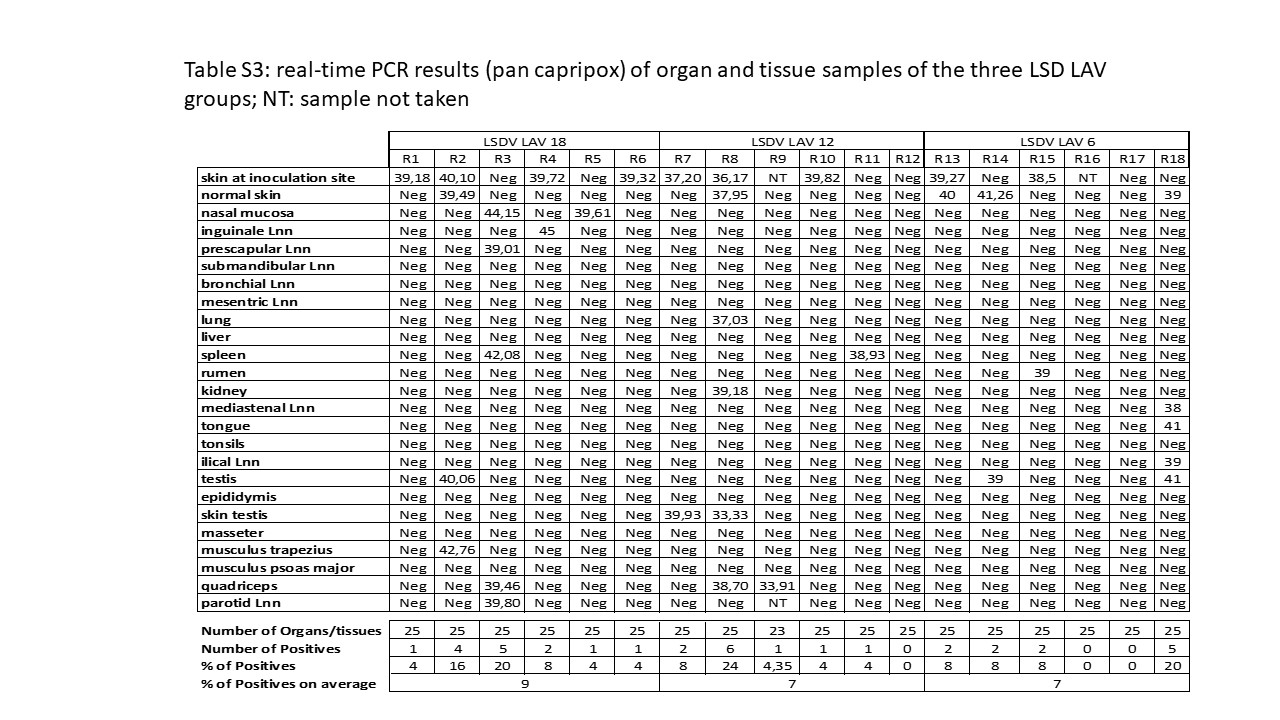

Supplement: Supplementary file 1 [file microorganisms-11-00210-s001.zip › Table S3_300dpi.jpg]

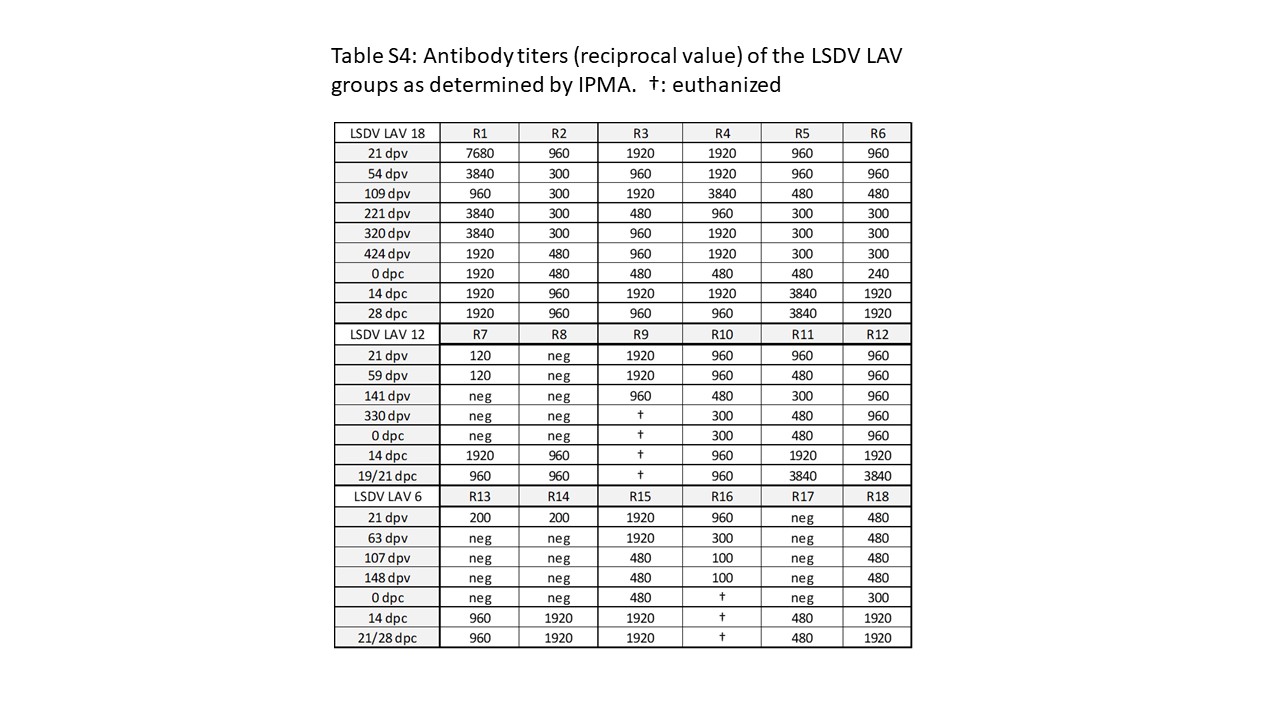

Supplement: Supplementary file 1 [file microorganisms-11-00210-s001.zip › Table S4_300dpi.jpg]

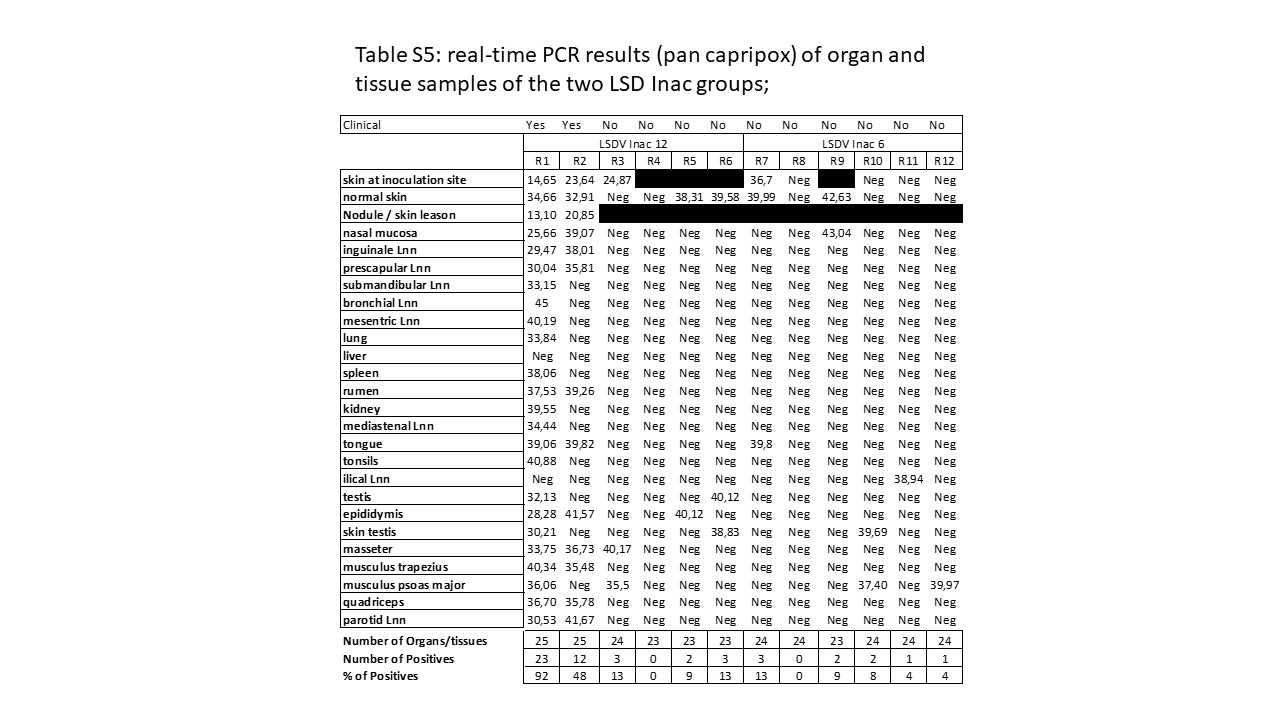

Supplement: Supplementary file 1 [file microorganisms-11-00210-s001.zip › Table S5_300dpi.jpg]

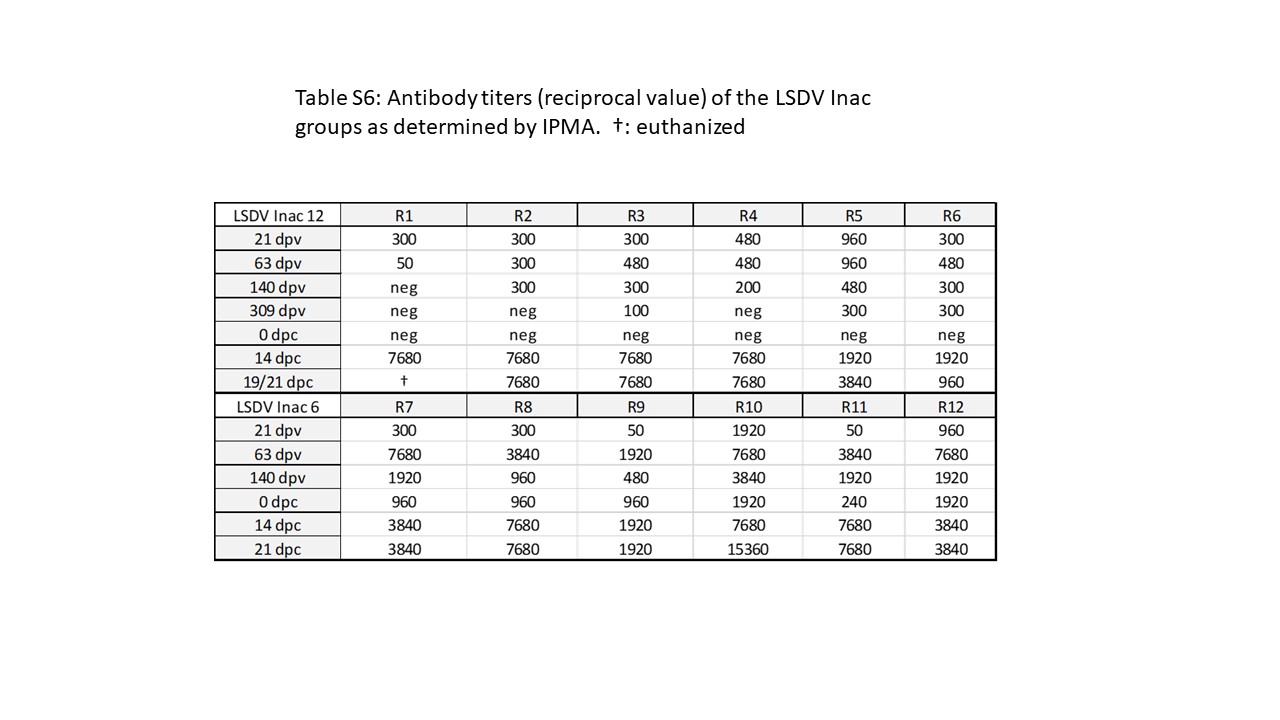

Supplement: Supplementary file 1 [file microorganisms-11-00210-s001.zip › Table S6_300dpi.jpg]
